# Superiority of a soft tissue‐based setup using cone‐beam computed tomography over a bony structure‐based setup in intensity‐modulated radiotherapy for prostate cancer^*^

**DOI:** 10.1120/jacmp.v16i5.5448

**Published:** 2015-09-08

**Authors:** Hiraku Sato, Eisuke Abe, Satoru Utsunomiya, Motoki Kaidu, Nobuko Yamana, Kensuke Tanaka, Atsushi Ohta, Mika Obinata, Junyang Liu, Gen Kawaguchi, Katsuya Maruyama, Fumio Ayukawa, Hidefumi Aoyama

**Affiliations:** ^1^ Department of Radiation Oncology Niigata University Graduate School of Medical and Dental Sciences Niigata Japan; ^2^ Department of Radiation Oncology Niigata University Medical and Dental Hospital Niigata Japan; ^3^ Department of Radiology Nagaoka Red Cross Hospital Nagaoka Japan

**Keywords:** intensity‐modulated radiotherapy (IMRT), prostate cancer, cone‐beam computed tomography (CBCT), image‐guided radiotherapy (IGRT), soft tissue‐based setup

## Abstract

The purpose of this study was to test the superiority of a soft tissue‐based setup using cone‐beam computed tomography (CBCT) to a bony structure‐based setup using the ExacTrac system in intensity‐modulated radiotherapy (IMRT) for prostate cancer. We studied 20 patients with localized prostate cancer who received IMRT between November 2010 and February 2012. After the initial setup, the pelvic bony structure‐based setup and ExacTrac system were applied. After that, CBCT and a soft tissue‐based setup were used. A shift in the isocenter between the ExacTrac‐based and CBCT‐based setup was recorded in the anterior–posterior (AP), superior–inferior (SI), and left–right (LR) axes. The shift was considered an interfractional prostate shift. Post‐treatment CBCT was also taken once a week to measure the intrafractional prostate shift, based on the coordinates of the isocenter between pre‐ and post‐treatment CBCT. The planning target volume (PTV) margins were determined using van Herk's method. We measured the elapsed time required for soft tissue matching and the entire treatment time using CBCT. The means±standard deviation(SD) of the inter‐ and intrafractional shifts were 0.9±2.8 mm and −0.3±1.4 mm in the AP, 0.9±2.2 mm and −0.1±1.2 mm in the SI, and 0.1±0.7 mm and −0.1±0.7 mm in the LR directions. The PTV margins in the cases of bony structure‐based and soft tissue‐based setups were 7.3 mm and 2.7 mm in the AP, 5.8 mm and 2.3 mm in the SI, and 1.9 mm and 1.2 mm in the LR directions. Even though the median elapsed time using CBCT was expanded in 5.9 min, the PTV margins were significantly reduced. We found the calculated PTV margins in the soft tissue‐based setup using CBCT were small, and this arrangement was superior to the bony structure‐based setup in prostate IMRT.

PACS numbers: 87.19.ru, 87.55.T‐

## I. INTRODUCTION

Intensity‐modulated radiotherapy (IMRT) has become a mainstay for the treatment of localized prostate cancer because IMRT techniques allow for dose escalation while minimizing toxicity to surrounding organs, such as the rectum and bladder.^1^, ^2^, ^3^, ^4^, ^5^, ^6^ Traditionally, patients with prostate cancer were positioned by using a combination of laser localization lights, skin tattoos, and a variety of body‐stabilizing devices. Portal images were acquired, and bony structure was used as a surrogate for the prostate position. However, it has been reported that the prostate moves in relation to the pelvic bony structure.^(7)^ Consequently, with improving technologies, more accurate image‐guided techniques using direct visualization of the prostate have been developed.

There are various image‐guided radiotherapy (IGRT) options to correct daily setup uncertainties and the positional variation of the prostate. The options include kV or MV portal imaging with fiducial markers (FMs),^(8)^ the ExacTrac system (BrainLAB, Heimstetten, Germany), B‐mode ultrasound (US),^(9)^ in‐room computed tomography (CT),^(10)^ various MV and kV cone‐beam CT (CBCT) techniques,^(11)^ and most recently, electromagnetic transponders.^(12)^

Interfractional setup errors can be reduced using image‐guided techniques. We show the superiority of a soft tissue‐based setup in reducing the planning target volume (PTV) margins over a bony structure‐based setup.

CBCT may better account for interfractional prostate motion than skin markers or bony structure and, therefore, it may reduce PTV margins. However, because a soft tissue‐based setup using CBCT takes a longer time compared to other IGRT options, it is likely to result in increased intrafractional prostate shifts, and it is necessary to expand the PTV margins. Budiharto and colleagues ^(13)^ reported that intrafractional prostate motion is increased by prolonged treatment times, and they noted that there would then be a need to expand the PTV margins.

In a CBCT‐based setup, the advantage of reduced interfractional prostate shifts and PTV margins might be offset by the disadvantages of increased intrafractional prostate shifts and PTV margins due to a prolonged treatment time. The superiority of a CBCT‐based setup compared to other IGRT options has thus remained unclear. In this study, we investigated that the calculated PTV margins in cases of a soft tissue‐based setup would be smaller than bony structure‐based setup, and the superiority of a soft tissue‐based setup using CBCT to a bony structure‐based setup in prostate IMRT was demonstrated.

## II. MATERIALS AND METHODS

The subjects were 20 patients with localized prostate cancer treated with IMRT at our hospital between November 2010 and May 2012. The patients' characteristics are summarized in Table 1. Each patient underwent CT simulation in the supine position with a customized vacuum immobilization device (Vac‐Lok; Med‐Tech, Orange City, IA) and with a filled bladder after evacuation. If more stools or gas than expected were found, the simulation was redone after evacuation or degassing using a Nelaton catheter. During the course we use a laxative when necessary.

All 20 patients were scanned on a 16‐slice CT scanner (LightSpeed RT, General Electric Medical Systems, Waukesha, WI) with the following clinical protocol: field of view (FOV) 40 cm, matrix 512×512, slice thickness 2.5 mm. IMRT plans were generated using an Eclipse treatment planning system ver. 8.9 (Varian Medical Systems, Palo Alto, CA). The prostate, seminal vesicles (SV), rectum, and bladder were delineated. In no SV invasion cases, the clinical target volume (CTV) consisted of the prostate and the proximal one‐third of the SV. In SV invasion cases, the CTV consisted of the prostate and the entire SV.

The margin of the CTV to the planning target volume (PTV) was 6 mm in the left–right, anterior, and superior directions and 5 mm in the posterior and inferior directions. Radiation was delivered with seven 6 MV coplanar beams on a Novalis‐TX system (Varian Medical Systems and BrainLAB, Feldkirchen, Germany). The first three patients received 70 Gy in 35 fractions five days/wk, the following fifteen received 70 Gy in 28 fractions four days/wk, and two patients with anticoagulation therapy received 67.5 Gy in 27 fractions four days/wk.

**Table 1:** Characteristics of the 20 patients

		*Data*
Age (y)	Median	65
	Range	55–77
Clinical stage (7^th^ UICC)	T1c	10
	T2a	5
	T2b	2
	T2c	1
	T3a	1
	T3b	1
Gleason score	≤6	3
	7	13
	≥8	4
Initial PSA (ng/mL)	Median	10.41
	Range	3.50–94.88
	Low	1
D'Amico risk group	Intermediate	14
	High	5

At treatment, the patients were initially positioned to the planning CT isocenter based on skin markers with a laser coordination system. After an initial setup, pelvic bony structure matching was carried out automatically using the ExacTrac X‐ray system and the matching was confirmed using the second ExacTrac image. This system was used to fuse a pair of oblique X‐ray images with digitally reconstructed radiographs (DRRs) obtained from simulation CT images within 1 mm shifts (in the anterior–posterior [AP], superior–inferior [SI], and left–right [LR] axes) and a $<1^{\circ }$ angle (pitch, roll, yaw).

Next, each of the patients underwent a daily pretreatment CBCT scan with the following clinical protocol: FOV 20 cm, matrix 512×512, slice thickness 1.0 mm, “full‐fan” acquisition. If more stools or gas than expected were found, the CBCT was redone after evacuation or degassing using a Nelaton catheter. The soft tissue matching was performed manually by radiation therapists using anatomic structures in the AP (the prostate–rectal interface, the anterior border of the prostate, and calcification), in the SI (the prostate–bladder border and calcification), and in the LR (the lateral borders of prostate and calcification), and then the matching was reviewed and approved by radiation oncologists. Post‐treatment CBCT was also conducted once a week to measure the intrafractional prostate shifts.

**Figure 1:**
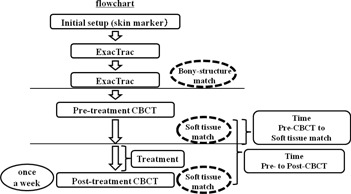
Flowchart of the study protocol.

A shift of the coordinates of the isocenter between the CBCT‐based setup and the bony structure‐based setup was recorded in the AP axis (i.e., the positive direction corresponds to the anterior from the planning isocenter), the SI axis (the positive direction corresponds to the superior), and the LR axis (the positive direction corresponds to the left). The shift was defined as an interfractional prostate shift on the basis of the pelvic bony structure. In our study, the interfractional prostate shifts using CBCT were assumed to be zero. A shift of the coordinates of the isocenter between the pre‐ and post‐treatment CBCT acquisition was also recorded. The shift was defined as an intrafractional prostate shift. The intrafractional prostate shifts in the bony structure‐based setup cannot be measured. Therefore, we assumed that the intrafractional prostate shifts in the bony structure‐based setup were same as those in the soft tissue‐based setup. We measured the time that elapsed between the pretreatment CBCT acquisition and the soft tissue matching, which indicates the time required for soft tissue matching. We also measured the elapsed time between the pre‐ and post‐treatment CBCT acquisitions, which indicates the entire treatment time for the soft tissue‐based setup using CBCT. A flowchart of the protocol is illustrated in Fig. 1.

We calculated the means and standard deviation (SD) of the interfractional and intrafractional prostate shifts. The random (σ) and systematic (Σ) errors for each setup were also calculated based on the inter‐ and intrafractional prostate shifts. We determined the PTV margins in each of the cardinal directions by van Herk's method (2.5Σ+0.7σ).^(14)^ To calculate the PTV margins in the soft tissue‐based setup, we assumed the interfractional prostate shifts to be zero.

All patients gave written informed consent concerning the use of their data for research purposes.

## III. RESULTS

For the analysis of the interfractional prostate shifts, a total of 577 pretreatment CBCTs were generated. The median number of scans per patient was 28 (range 27–35). For the analysis of the intrafractional prostate shifts, a total of 131 post‐treatment CBCTs were generated. The number of patients who received post‐treatment CBCTs was 19. The data of the intrafractional prostate shifts of 19 patients were acquired. The median number of scans per patient was seven (range six to eight). The means ±SD of the interfractional prostate shifts in the 577 pretreatment CBCTs on the basis of the pelvic bony structure were 0.9±2.8 mm in the AP axis, 0.9±2.2 mm in the SI axis, and 0.1±0.7 mm in the LR axis. The means ±SD of the intrafractional prostate shifts in the 131 pre‐ and post‐treatment CBCTs were −0.3±1.4 mm in the AP axis, −0.1±1.2 mm in the SI axis, and −0.1±0.7 mm in the LR axis.

Histograms of the inter‐ and intrafractional prostate shifts are given in 2, 3. The Σ and σ values of the inter‐ and intrafractional prostate shifts are listed in Table 2. The PTV margins calculated by van Herk's method (2.5Σ+0.7σ)^(14)^ in the bony structure‐based setup using the ExacTrac system and the PTV margins calculated by the same method in the soft tissue‐based setup using CBCT are given in Table 3. The median elapsed time required for soft tissue matching was 5.9 min (range 2.8–13.6 min), and the median entire treatment time in the soft tissue‐based setup using CBCT was 12.5 min (range 9.0–22.0 min).

**Figure 2:**
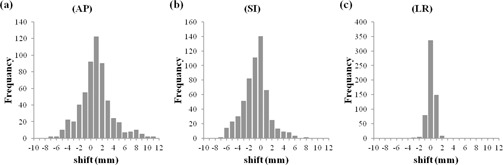
Histograms of interfractional prostate shifts; the total number of shifts using pretreatment CBCT were 577: (a) the AP axis — the positive direction indicates the anterior position from the planning isocenter; (b) the SI axis — the positive direction indicates the superior position; (c) the LR axis — the positive direction indicates the left position.

**Figure 3:**
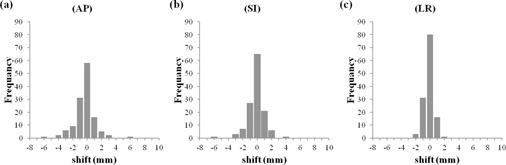
Histograms of intrafractional prostate shifts; the total number of post‐treatment CBCT images were 131: (a) the AP axis — the positive direction indicates the anterior position from the planning isocenter; (b) the SI axis — the positive direction indicates the superior position; (c) the LR axis — the positive direction indicates the left position.

**Table 2:** Σ and σ values of the inter‐ and intrafractional prostate shifts

*Interfractional Prostate Shifts of 20 Patients in Each Direction (mm)*				*Intrafractional Prostate Shifts of 19 Patients in Each Direction (mm)*			
	*AP*	*SI*	*LR*		*AP*	*SI*	*LR*
$\Sigma_{\text{inter}}$	2.2	1.7	0.4	$\Sigma_{\text{intra}}$	0.7	0.6	0.3
$\sigma_{\text{inter}}$	1.8	1.5	0.6	$\sigma_{\text{intra}}$	1.3	1.1	0.7

$\Sigma_{\text{inter}}=\text{SD of the systematic errors of the interfractional prostate shifts}$.

$\sigma_{\text{inter}}=\text{Root mean square of the random errors of the interfractional prostate shifts}$

$\Sigma_{\text{intra}}=\text{SD of the systematic errors of the intrafractional prostate shifts}$.

$\sigma_{\text{intra}}=\text{Root mean square of the random errors of the intrafractional prostate shifts}$.

**Table 3:** PTV margins in the bony structure‐based and soft tissue‐based setups

*PTV Margins in Bony Structure‐Based Setup (mm)*				*PTV Margins in Soft Tissue‐Based Setup (mm)*			
	*AP*	*SI*	*LR*		*AP*	*SI*	*LR*
2.5Σ+0.7σ	7.3	5.8	1.9	2.5Σ+0.7σ	2.7	2.3	1.2

$\sum =\sqrt{{\sum_{inter}}^{2}+{\sum_{intra}}^{2}}$

$\sigma =\sqrt{{\sigma_{inter}}^{2}+{\sigma_{intra}}^{2}}$

## IV. DISCUSSION

In our study, the intrafractional prostate shifts were similar to those in previous studies. Budiharto and colleagues ^(13)^ reported that the mean±SD of the intrafractional prostate shifts detected using FMs were 2.3±1.5 mm in the AP axis, 0.2±1.1 mm in the SI axis, and −0.1±1.1 mm in the LR axis. Huang and colleagues ^(15)^ reported that the mean±SD of the intrafractional shifts detected using a B‐mode US system were 0.2±1.3 mm in the AP axis, 0.1±1.0 mm in the SI axis, and 0.01±0.4 mm in the LR axis.

If intrafractional prostate shifts are increased, the PTV margins in both the soft tissue‐based and bony structure‐based setups are increased. Since the soft tissue‐based setup using CBCT took additional time to manually match anatomic structure, the PTV margins became larger as elapsed time increased in this setup. However, the PTV margins in the soft tissue‐based setup were small (Table 3).

If interfractional prostate shifts are increased, the differences of the PTV margins between the bony structure‐based and soft tissue‐based setup should be increased. The data in Table 3 show that the PTV margins in the soft tissue‐based setup were smaller compared to those in the bony structure‐based setup, particularly in the AP direction. These results indicate that the corrections of interfractional prostate shifts were greatly important. In addition, as the interfractional prostate shifts in the AP direction were greatly dependent on the quantity of rectal contents, there was a limit in the bony structure‐based setup. This limit was extremely effective for reduction of the PTV margins, particularly in the AP direction, in the soft tissue‐based setup using CBCT compared to the bony structure‐based setup.

Although the PTV margins calculated using IGRT were reduced, we still should be careful in reducing the clinically used margins. Engels and colleagues ^(16)^ reported that the five‐year freedom from biochemical failure rate in patients positioned using FMs and PTV margins of 5 mm in the AP and SI axes and 3 mm in the LR axis was 58%, which is significantly lower than the 91% obtained in the cases without FMs (p=0.02). They proposed that a reduction of PTV margins should be done carefully because margins that are too‐narrow may result in local control failure. We note that we do not simply use the calculated CTV to PTV margin in creating clinical treatment plans.

It was reported that intrafractional prostate shifts were increased with the extension of the treatment time. Mah and colleagues ^(17)^ quantified the intrafractional prostate shifts in 9 min with cine‐MRI studies. The means ±SD were 0.2±2.9 mm in the AP axis, 0.0±3.4 mm in the SI axis, and 0.0±1.5 mm in the LR axis. Budiharto and colleagues ^(13)^ obtained relevant information on the prostate's position at 30‐sec intervals during IMRT, and they emphasized the need to speed up the mark‐match procedure, as the intrafractional prostate shifts were increased with longer elapsed times. They reported that the SD of the intrafractional prostate shifts at 10min was increased to 3.5 mm.

CBCT acquisition takes additional time compared to other IGRT procedures. It would thus be necessary to greatly increase the PTV margins with the increase of the intrafractional prostate shifts when there are longer elapsed times between pretreatment CBCT acquisition and soft‐tissue matching.

Our intrafractional prostate shifts in 12.5 min were not greatly different compared to the results reported by Mah and colleagues,^(17)^ in which internal prostate shifts were measured in 9 min, even though our elapsed time was longer than theirs. The reason for this might be due to differences in setup accuracy or the immobilization device.

One of the limitations of this study is that the quality of the CBCT images was not perfect for the consistent, reproducible identification of borders of the prostate. In addition, there were interobserver and intraobserver variations in the soft‐tissue matching using CBCT. In order to obtain fairly consistent soft‐tissue matching accuracy among the patients, it might be effective to use FMs and to match automatically. However, since the soft tissue‐based setup using CBCT was a noninvasive procedure, it is appropriate for patients with systemic complications and those who refuse implantation.

As noted above, we did not recognize the real‐time internal prostate motion between pre‐ and post‐treatment CBCT, and we did not measure the intrafractional prostate shifts in cases in which the bony structure‐based setup was used.

## V. CONCLUSIONS

We verified that the calculated PTV margins in a soft tissue‐based setup were small, and we observed the superiority of the soft tissue‐based setup compared to a bony structure‐based setup in prostate IMRT. Although the soft tissue‐based setup using CBCT took additional time, its highly accurate alignment may compensate for the disadvantage of prolonged treatment time.

## ACKNOWLEDGMENTS

This work was supported by the Funding Program for World‐Leading Innovative R&D on Science and Technology (FIRST Program) initiated by the Council for Science and Technology Policy, a Grant‐in‐Aid for Scientific Research (21591602) from the Japanese Society for the Promotion of Science, and a Grant‐in‐aid from the Niigata University Science Foundation.
